# A dual-stage partially interpretable neural network for joint suppression of bSSFP banding and flow artifacts in non-phase-cycled cine imaging

**DOI:** 10.1186/s12968-023-00988-z

**Published:** 2023-11-23

**Authors:** Zhuo Chen, Sha Hua, Juan Gao, Yanjia Chen, Yiwen Gong, Yiwen Shen, Xin Tang, Yixin Emu, Wei Jin, Chenxi Hu

**Affiliations:** 1 National Engineering Research Center of Advanced Magnetic Resonance Technologies for Diagnosis and Therapy, School of Biomedical Engineering, Shanghai Jiao Tong University, 415 S Med-X Center, 1954 Huashan Road, Shanghai, 200030 China; 2 Department of Cardiovascular Medicine, Heart Failure Center, Ruijin Hospital Lu Wan Branch, Shanghai Jiao Tong University School of Medicine, Shanghai, China

**Keywords:** Artifact, SSFP, Flow, Banding, Deep learning, Interpretable

## Abstract

**Purpose:**

To develop a partially interpretable neural network for joint suppression of banding and flow artifacts in non-phase-cycled bSSFP cine imaging.

**Methods:**

A dual-stage neural network consisting of a voxel-identification (VI) sub-network and artifact-suppression (AS) sub-network is proposed. The VI sub-network provides identification of artifacts, which guides artifact suppression and improves interpretability. The AS sub-network reduces banding and flow artifacts. Short-axis cine images of 12 frequency offsets from 28 healthy subjects were used to train and test the dual-stage network. An additional 77 patients were retrospectively enrolled to evaluate its clinical generalizability. For healthy subjects, artifact suppression performance was analyzed by comparison with traditional phase cycling. The partial interpretability provided by the VI sub-network was analyzed via correlation analysis. Generalizability was evaluated for cine obtained with different sequence parameters and scanners. For patients, artifact suppression performance and partial interpretability of the network were qualitatively evaluated by 3 clinicians. Cardiac function before and after artifact suppression was assessed via left ventricular ejection fraction (LVEF).

**Results:**

For the healthy subjects, visual inspection and quantitative analysis found a considerable reduction of banding and flow artifacts by the proposed network. Compared with traditional phase cycling, the proposed network improved flow artifact scores (4.57 ± 0.23 vs 3.40 ± 0.38, P = 0.002) and overall image quality (4.33 ± 0.22 vs 3.60 ± 0.38, P = 0.002). The VI sub-network well identified the location of banding and flow artifacts in the original movie and significantly correlated with the change of signal intensities in these regions. Changes of imaging parameters or the scanner did not cause a significant change of overall image quality relative to the baseline dataset, suggesting a good generalizability. For the patients, qualitative analysis showed a significant improvement of banding artifacts (4.01 ± 0.50 vs 2.77 ± 0.40, P < 0.001), flow artifacts (4.22 ± 0.38 vs 2.97 ± 0.57, P < 0.001), and image quality (3.91 ± 0.45 vs 2.60 ± 0.43, P < 0.001) relative to the original cine. The artifact suppression slightly reduced the LVEF (mean bias = -1.25%, P = 0.01).

**Conclusions:**

The dual-stage network simultaneously reduces banding and flow artifacts in bSSFP cine imaging with a partial interpretability, sparing the need for sequence modification. The method can be easily deployed in a clinical setting to identify artifacts and improve cine image quality.

**Supplementary Information:**

The online version contains supplementary material available at 10.1186/s12968-023-00988-z.

## Introduction

Balanced steady state free precession (bSSFP) cine is the major cine sequence in clinical imaging due to its high signal to noise ratio (SNR) and contrast to noise ratio (CNR) [1, 2]. However, two types of artifacts—banding artifacts and flow artifacts—are common in bSSFP cine and may severely degrade the image quality [3]. Banding artifacts appear as dark bands in the image, and can be reduced by shimming, frequency scout, and phase cycling [4–15]. Among them, phase cycling is highly effective, but it also increases the scan time and has received limited clinical use. Furthermore, phase cycling often shifts the off-resonance into the flow regions, inducing new flow artifacts that would be otherwise absent. Flow artifacts can be induced by several causes, among which the most common one is the out-of-slice signals of spins flowing through a dark band. In this case, flow artifacts often cause spurious hyperenhancement along the phase-encoding direction and obscure neighboring tissues [16–18]. Flow artifacts can be suppressed by flow-compensation gradients [19], partial dephasing [20, 21], and slice-encoding [22].

Although the methods above can suppress the two artifacts, most of them require sequence modifications [23]. On the other hand, deep learning-based post-processing methods have received little attention for this application despite its outstanding performance in suppression of other artifacts [24–29]. Thus far, deep learning has only been applied to suppress banding artifacts in non-cine bSSFP imaging [30]. Removing banding artifacts for bSSFP cine imaging is more challenging because acquisition of cine movie labels free of both banding and flow artifacts is more difficult. Moreover, deep learning is often criticized for its lack of interpretability [31]. When artifacts are removed, whether the removal was truly based on recognition of the artifacts often remains unknown for a simple neural network.

In this study, we sought to develop a partially interpretable dual-stage network to jointly suppress banding and flow artifacts in non-phase-cycled bSSFP cine imaging (i.e. regular bSSFP cine). Since the method does not require acquisition of phase-cycled data in the testing stage, the method is a post-processing technique. Interpretability of the network was improved by using two cascaded U-Nets, in which the first U-Net [32] recognizes the location and type of artifacts, and the second U-Net suppresses the artifacts with the guidance of the first U-Net. The dual-stage network was trained using a phase-cycling method tailored to improve the balance between suppression of banding artifacts and promotion of flow artifacts. Evaluation was performed with both healthy subjects and patients using a variety of sequence parameters.

## Materials and methods

### Imaging data

Imaging was performed in both healthy subjects and patients, who were prospectively enrolled from a research institution and retrospectively enrolled from a hospital, respectively. The healthy-subject data was used to train and test the proposed neural network with different sequence parameters. The patient data was used to evaluate generalizability of the network for a different scanner and cohort. The study was approved by the institutional review board from each participating institution. All healthy subjects and patients provided written informed consent prior to the scan.

#### Baseline dataset

Twenty-eight healthy subjects (10 male, age 24 ± 2 years) were imaged with a bSSFP cine sequence in a 3T scanner (uMR 790, United Imaging Healthcare, Shanghai, China) with a 12-channel torso coil and 32-channel spine coil. For each subject, a retrospectively gated cine sequence was performed in three short-axis slices located in the apex, mid-ventricle, and base of the left ventricle (LV). For each slice, the sequence was repeated 12 times, each with a different center frequency. The resultant center frequencies uniformly covered the off-resonance frequency range between -1/(2*TR) and 1/(2*TR). Sequence parameters are noted in Table 1.

**Table 1:** Acquisition parameters for each dataset

Key characteristics	Baseline dataset	Generalization dataset				Clinical dataset
	Baseline parameters	Altered bandwidth	Altered flip angle	Altered slice thickness	Altered views	A different scanner
Type of subjects	Healthy subjects	Healthy subjects	Healthy subjects	Healthy subjects	Healthy subjects	Patients
Number of subjects	28	11	11	11	11	77
View	Sax	SAx	SAx	SAx	2-ch, 4-ch	Sax, 2-ch, 4-ch
FOV (RO × PE, mm)	360 × 320	360 × 320	360 × 320	360 × 320	360 × 320	360 × 320 or 380 × 340
Matrix size (RO × PE)	336 × 298	336 × 298	336 × 298	336 × 298	336 × 298	336 × 298 or 336 × 300
Number of slices	3	1	1	1	1, 1^a^	9–15, 1, 1^b^
Slice thickness (mm)	8	8	8	5	8	8
Number of cardiac phases	25	25	25	25	25	25
TR (msec)	2.86 or 2.98	3.62 or 3.71	2.86 or 2.98	3.15 or 3.17	2.86 or 2.98	3.03–3.10
TE (msec)	1.31 or 1.37	1.60 or 1.66	1.31 or 1.37	1.46 or 1.44	1.31 or 1.37	1.42–1.46
Bandwidth (Hz/pixel)	1000	500	1000	1000	1000	1000
Flip angle (°)	60	60	30	60	60	44–60
Frequency offsets (Hz)	± 137.5, ± 110, ± 82.5, ± 55, ± 27.5, 0, 165	0, 55, 110, 165	0, 55, 110, 165	0, 55, 110, 165	0, 55, 110, 165	0
Total number of movies	1008	44	44	44	44, 44	905, 77, 77

Sax, short-axis; 2-ch,  2-chamber; 4-ch,  4-chamber; TR,  repetition time; TE,  echo time; RO,  readout; PE,  phase-encoding

^a^1,1 indicates the number of slices for 2-ch and 4-ch is 1 and 1, respectively

^b^9–15,1,1 indicates the number of slices for short-axis, 2-ch and 4-ch is 9–15, 1 and 1, respectively

#### Generalization dataset

Eleven healthy subjects (4 males, age 24 ± 2 years) were also imaged with altered sequence parameters to evaluate generalizability of the method. A total of 4 carefully chosen parameters, including the bandwidth, flip angle, slice thickness, and imaging views, were individually altered in the experiment. The bandwidth was changed from the baseline 1000 Hz to 500 Hz, which incurred a longer TR and thus more banding artifacts. The flip angle was changed from the baseline 60° to 30°, which damped the myocardium-to-blood contrast and changed the intensity distribution. The slice thickness was changed from the baseline 8 mm to 5 mm, which caused stronger flow artifacts. The imaging view was changed from the baseline short-axis view to 2-chamber and 4-chamber views, which changed the image appearance. For the generalization dataset, only 4 center frequencies uniformly covering the frequency range between −1/(2*TR) and 1/(2*TR) were imaged. Other parameters are noted in Table 1.

#### Clinical dataset

A total of 77 patients (53 male, age 50 ± 17 years) consecutively scanned between July 2022 and February 2023 in the Ruijin-Luwan Hospital were retrospectively enrolled. All patients were scanned on a 3T clinical scanner (uMR 890, United Imaging Healthcare, Shanghai, China) with a 12-channel torso and 32-channel spine coil. Clinical indications included ischemic cardiomyopathies (n = 2), nonischemic cardiomyopathies (n = 47), arrhythmia (n = 4), hypertension (n = 3), heart failure (n = 3), and physical examination (n = 18). Retrospectively gated bSSFP cine imaging was performed in 9–15 short-axis slices covering the whole LV and 2 long-axis slices (2-chamber and 4-chamber). Other sequence parameters are noted in Table 1.

### Dual-stage partially-interpretable neural network

#### Architecture

**Fig. 1:**
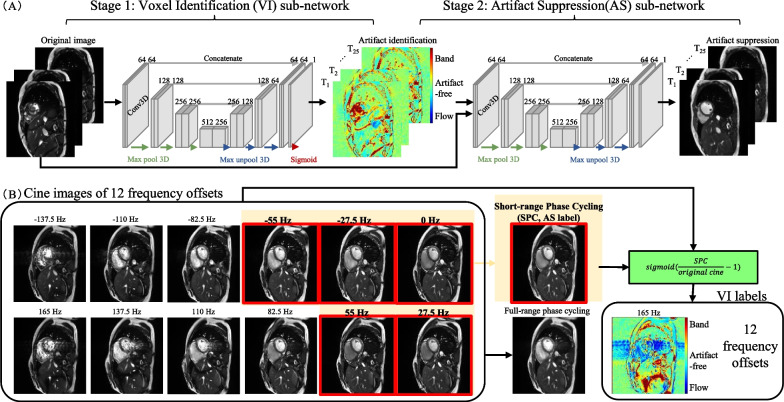
Schematic of the dual-stage network and generation of the training labels. **A** The dual-stage network consists of VI and AS sub-networks. Both sub-networks use a 3-dimensional U-Net as the backbone. The output from the VI sub-network provides a soft classification of banding and flow artifacts, which is used to guide artifact suppression by the AS sub-network and improve interpretability of the network. **B** Twelve cine movies each with a different center frequency offset were obtained. Average of the movies with 5 centric frequency offsets (red boxes) generated the label for training of the AS sub-network. The corresponding method was called “short-range phase cycling (SPC)”. The label for the VI sub-network was obtained through the equation $sigmoid(SPC\_label/original\_cine-1)$ based on the SPC-combined image and the original cine. Since banding artifacts are dark and flow artifacts in concern of this paper are bright, this equation generates an intrinsic label for the VI sub-network

Figure 1A shows a schematic of the proposed neural network. The network comprises of two sub-networks: a voxel identification (VI) sub-network to identify the artifacts and an artifact suppression (AS) sub-network to suppress the artifacts. Both sub-networks use a 3-dimensional U-Net architecture as the backbone [32], with nearly the same architecture except for the input and output layers. The VI sub-network takes the original cine movie as input and outputs a voxel-identity map (VI map), which has a value near 1 if the underlying voxel is in a dark band, near 0 if in a flow artifact, and around 0.5 if in an artifact-free zone. The AS sub-network takes both the original cine movie and the corresponding VI map as the inputs, and outputs an artifact-suppressed cine movie. Additional file 1 provides a detailed explanation of the architecture used by both VI and AS sub-networks.

#### Generation of training labels

Training of the dual-stage network requires cine movies free of both banding and flow artifacts. In this work, we use phase cycling to generate these cine movie labels, as illustrated in Fig. 1B. However, to avoid incurrence of flow artifacts, we developed a short-range phase cycling (SPC) method, which only averaged cine movies collected at 5 central frequency offsets (0, ± 27.5, and ± 55 Hz). Since these frequencies typically do not incur banding artifacts in the heart, the risk of flow-artifact incurrence is considerably reduced. On the other hand, inclusion of the 5 central frequencies covered nearly ½ of the full phase-cycling range and thus their linear combination considerably reduces the banding artifacts. The proposed SPC thus achieves a better balance between suppression of banding and flow artifacts in comparison with traditional phase cycling, which cycles through the entire range of frequencies. The latter was termed “full-range phase cycling” (FPC) and compared with the SPC-trained network in our experiment.

Once the SPC label was obtained, the training label for the VI sub-network was generated by $\mathrm{sigmoid}(\mathrm{SPC}\_\mathrm{label}/\mathrm{original}\_\mathrm{cine}-1)$. This formula ensures that the resultant image has an intensity between 0 and 1. Furthermore, since dark bands are dark and flow artifacts in concern of this paper are bright, their corresponding intensities after these operations are near 1 and 0, respectively, providing an intrinsic identification of these two artifacts.

#### Training

Short-axis cine movies collected from 18 healthy subjects were used for training, generating a training dataset of 648 (18 subjects × 3 slices × 12 frequency offsets) movies. Movies from the remaining 10 healthy subjects in the baseline dataset were used for testing. Details of the pre-processing steps and training parameters are found in Additional file 1. Note that the VI sub-network was trained before the AS sub-network. During training of the AS sub-network, the VI sub-network was frozen and only used to provide the VI map.

### Evaluation

Evaluation of the proposed method comprised of several experiments, which are briefly described blow. More details about the evaluation are found in Additional file 1.

#### Artifact suppression performance

The performance of artifact suppression was verified by comparing the averaged signal of the network output in the heart over 12 frequency offsets. If artifacts were suppressed, the averaged signal should be nearly constant over different frequencies. The accuracy of artifact identification by the VI sub-network was verified by comparing its output with the training label in the heart.

#### Comparison with FPC

The proposed network was further compared with FPC in terms of banding artifacts, flow artifacts, and overall image quality to see if there exists any advantage. The comparison was based on qualitative scoring using a 5-point Likert scale (1: non-diagnostic; 2: poor; 3: fair; 4: good; and 5: excellent) from 3 readers (CH with 8 years of CMR experience, JG and XT with 4 years of CMR experience). Each reader performed the evaluation independently and blindly, and their scores were averaged for the final assessment.

#### Partial interpretability

To investigate the consistency between the VI map and the AS output, we performed a correlation analysis between the VI map and $\mathrm{sigmoid}(\mathrm{AS}\_\mathrm{output}/\mathrm{original}\_\mathrm{cine}-1)$ for the mid-ventricle slice of each subject in the testing dataset. A correlation between the two variables indicates that an observation of high or low values in the VI map predicts a modification of the input image at the same place.

#### Generalizability

Generalizability of the dual-stage network was evaluated by comparing the network output between different imaging groups, including the testing dataset (baseline), the reduced-bandwidth dataset (BW), the reduced-flip-angle dataset (FA), the reduced-slice-thickness dataset (ST), the long-axis dataset (LAx), and the clinical dataset (Clin). The data across all groups were merged together and the evaluation was unpaired. Qualitative evaluations were performed by the same readers (CH, JG, and XT) blindly and independently, according to the same 5-point Likert scale. Their scores were averaged for the final assessment.

#### Clinical evaluations

Performance of the dual-stage network for the clinical dataset was evaluated by three clinicians, including two cardiologists and a technologist (SH, YG, and YS, with 4, 3, and 4 years of CMR experience, respectively). Forty-eight patients with relatively severe image artifacts in the original cine movie were chosen by an observer blinded to the network output. For each patient, the original movie, the VI map, and the AS output image were reviewed by each clinician. The clinicians were not blinded to the identity of each movie, because of their large difference in appearance and the need to evaluate the partial interpretability. Banding artifact suppression, flow artifact suppression, and overall image quality were scored using the 5-point Likert scale. Identification of banding and flow artifacts by the VI sub-network was scored using a two-point scale (1: the VI map does not help identification of the artifacts; and 2: the VI map helps identification of the artifacts).

The LV ejection fraction (LVEF) was quantified in the 77 patients for both the original short-axis cine movies and the artifact-suppressed movies. The assessment aimed to show whether the artifact suppression caused any clinically significant changes of LVEF.

### Statistical analysis

The Wilcoxon signed-rank test was used to evaluate differences in paired qualitative comparisons and Wilcoxon rank-sum test in unpaired qualitative comparisons. Paired t-test and Bland–Altman plots were used to evaluate the LVEF difference between the original and artifact-suppressed movies. The inter-observer agreement was evaluated by intraclass correlation coefficient (ICC). P < 0.05 was considered statistically significant. Statistical analyses were performed using IBM SPSS Statistics (version 27.0, IBM, Armonk, New York, USA).

## Results

### Artifact suppression performance

**Fig. 2:**
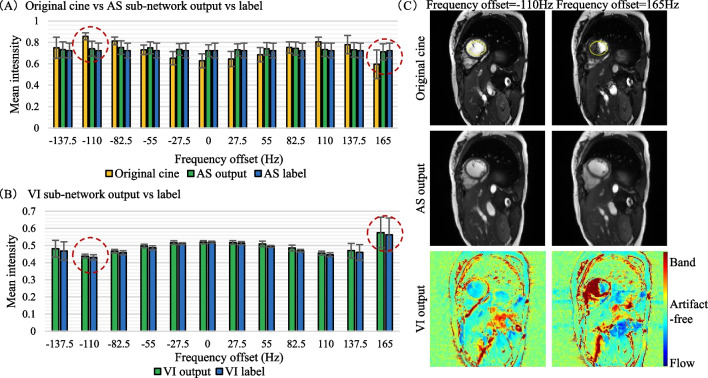
Performance of the dual-stage network in the multi-frequency testing dataset. **A** The mean intensities of the original cine, AS sub-network output, and AS sub-network label in the end-diastolic heart. Consistent with the labels, the AS output showed considerably smaller variations and relatively constant mean signals across the 12 frequency offsets. **B** The mean values of VI sub-network output was consistent with the mean values of the VI sub-network label in the end-diastolic heart. Furthermore, the VI outputs had an anti-correlated variation relative to the mean intensity of the original cine images, suggesting a good classification of the artifact categories by the VI sub-network. **C** Two representative examples with a frequency offset of − 110 Hz and 165 Hz, respectively. ROIs were circled in yellow. AS, artifact suppression; VI, voxel identification

In the testing dataset, the original cine exhibited a periodic signal variation in the heart over 12 frequency offsets due to the presence of banding or flow artifacts. The AS sub-network output showed considerably smaller variations over the 12 frequencies and a higher consistency with the label (Fig. 2A). The VI sub-network output and its label exhibited a consistent trend (Fig. 2B), which was anti-correlated with the mean intensity of the original cine, suggesting that the VI sub-network correctly classified the artifact category in the heart. Two representative examples are shown in Fig. 2C.

### Comparison with FPC

**Fig. 3:**
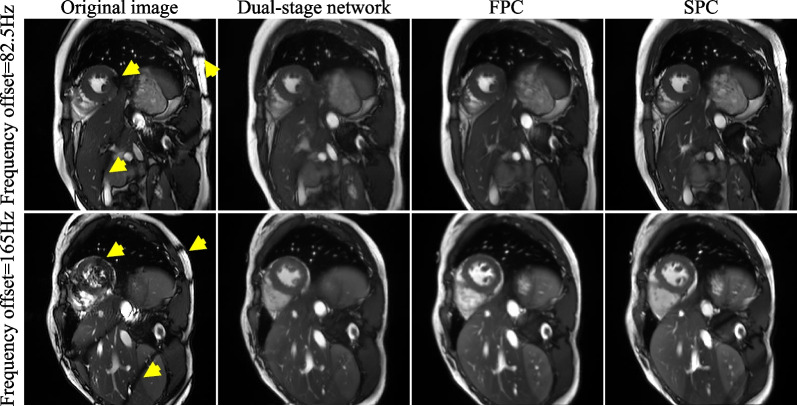
A representative example of banding artifact suppression by the proposed network, FPC, and SPC at two different frequencies. Banding artifacts appeared in the heart, abdomen, and subcutaneous fat regions (yellow arrows). All three methods suppressed these banding artifacts. FPC, full-range phase cycling; SPC, short-range phase cycling

**Fig. 4:**
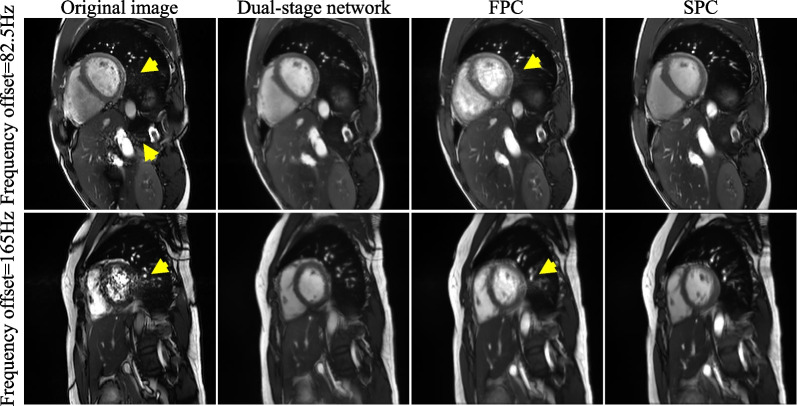
A representative example of flow artifact suppression by the proposed network, FPC, and SPC at two different frequencies. Flow artifacts appeared in the heart and abdomen regions (yellow arrows). Both the dual-stage network and SPC well reduced flow artifacts. On the other hand, FPC still resulted in considerable flow artifacts in the heart region. FPC, full-range phase cycling; SPC, short-range phase cycling

**Fig. 5:**
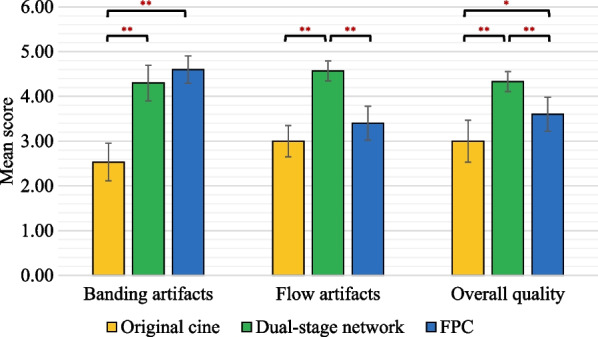
Qualitative comparisons of the original cine movie, the dual-stage network output, and FPC output by a 5-point Likert scale (5 is the best). The dual-stage network and FPC reduced banding artifacts compared to the original cine. The dual-stage network improved flow artifacts compared to both the original cine and FPC. Both the dual-stage network and FPC improved image quality than the original cine. Furthermore, the dual-stage network yielded a better image quality than FPC. FPC, full-range phase cycling

Figures 3 and 4 show exemplary suppressions of the banding and flow artifacts by the dual-stage network, FPC, and SPC at two frequencies. All three methods well suppressed the banding artifacts relative to the original images (Fig. 3). However, only the dual-stage network and SPC well suppressed the flow artifacts, whereas FPC did not (Fig. 4). The qualitative analysis (Fig. 5) shows that both the network and FPC achieved banding artifact scores of 4.30 ± 0.40 and 4.60 ± 0.31, respectively, higher than that of the original cine (2.53 ± 0.42). However, for flow artifacts, the dual-stage network achieved higher scores than both the original cine (4.57 ± 0.23 vs 3.00 ± 0.35, P = 0.002) and FPC (3.40 ± 0.38, P = 0.002). Furthermore, the overall quality of the dual-stage network was significantly higher than the original cine (4.33 ± 0.22 vs 3.00 ± 0.47, P = 0.002) and FPC (3.60 ± 0.38, P = 0.002), likely due to the better suppression of flow artifacts. ICCs of the 3 readers were 0.87 (95% CI [0.64, 0.94]) for banding artifacts, 0.60 (95% CI [0.28, 0.80]) for flow artifacts, and 0.58 (95% CI [0.16, 0.80]) for overall image quality.

### Partial interpretability

**Fig. 6:**
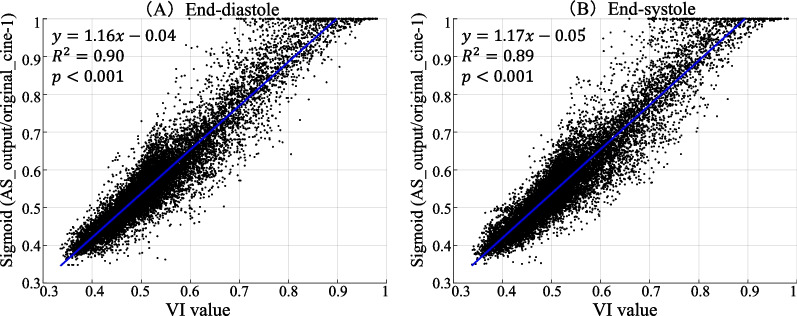
Results of the voxel-level correlation between the VI output and image modification evaluated by $\mathrm{sigmoid}(\mathrm{AS}\_\mathrm{output}/\mathrm{original}\_\mathrm{cine}-1)$ at both end-diastole (**A**) and end-systole (**B**) of a single subject. The two variables significantly correlated with each other. VI, voxel identification; AS, artifact suppression

Figure 6 shows the significant correlations between voxelwise VI values and image modifications represented by $\mathrm{sigmoid}(\mathrm{AS}\_\mathrm{output}/\mathrm{original}\_\mathrm{cine}-1)$ for a single subject. The original cine, VI output, and AS output movies of this subject are shown in Additional file 2. This correlation was significant for every subject in the testing dataset (P < 0.001; ${R}^{2}$= 0.89 ± 0.02 for end-diastole and 0.89 ± 0.01 for end-systole). The results suggest that the VI values can be used to interpret the modification of the input cine image by the dual-stage network.

### Generalizability

**Fig. 7:**
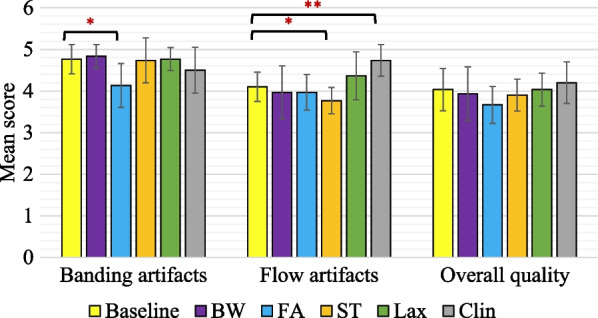
The results of unpaired statistical comparisons between the baseline dataset (baseline), which had the same sequence parameters with the training dataset, and other datasets, which had different sequence parameters. The change of sequence parameters included reduced bandwidth (BW), reduced flip angles (FA), reduced slice-thickness (ST), use of long-axis views (Lax), and use of a clinical scanner for imaging of patients (Clin). Except the FA group, the banding artifact suppression was not significantly different compared with the baseline. Except the Lax and Clin groups, the flow artifact suppression was not significantly different compared with the baseline. The overall image quality for all groups of changed parameters was not significantly different compared with the baseline. These results suggest that the proposed network was reasonably generalizable when relevant sequence parameters were changed

**Fig. 8:**
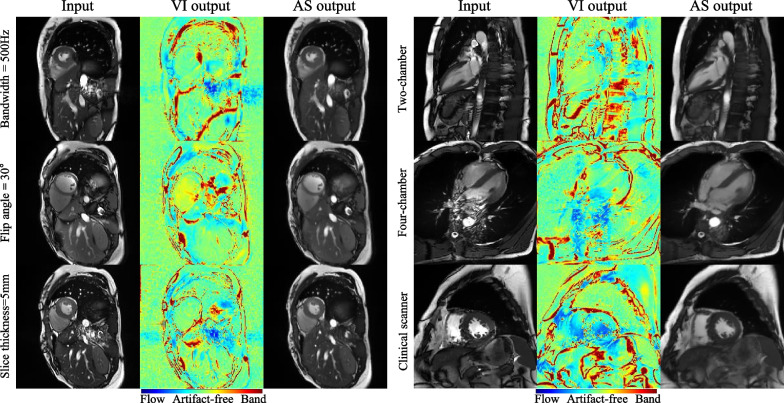
Six representative cine images and their corresponding VI sub-network outputs and AS sub-network outputs for different imaging parameters relative to the baseline dataset. Banding artifacts in the heart, abdomen, and subcutaneous fat, and the flow artifacts in the aorta were well suppressed by the dual-stage network, even though the latter was not trained with these imaging parameters, views, or scanner. The VI maps generated by the VI sub-network identified the location and type of each artifact, rendering the correction results interpretable. VI, voxel identification; AS, artifact suppression

Compared with the baseline (4.77 ± 0.35), changes of bandwidth (4.83 ± 0.28, P = 0.69), slice-thickness (4.73 ± 0.54, P = 0.79), imaging view (4.77 ± 0.27, P = 0.83), and scanner (4.50 ± 0.55, P = 0.21) did not cause significant changes of banding artifacts, whereas reduction of flip angle to 30° slightly increased the banding artifacts (4.13 ± 0.53, P = 0.01) (Fig. 7). For the flow artifacts, changes of bandwidth (3.97 ± 0.64, P = 0.37), flip angle (3.97 ± 0.43, P = 0.47), and imaging view (4.37 ± 0.58, P = 0.20) did not significantly change the scores, whereas decreased slice thickness caused increased flow artifacts (3.77 ± 0.32, P = 0.03), and the clinical scanner caused decreased flow artifacts (4.73 ± 0.38, P = 0.003). Finally, the overall image quality was not significantly changed for every generalization dataset (BW: 3.93 ± 0.64, P = 0.40; FA: 3.67 ± 0.44, P = 0.10; ST: 3.90 ± 0.39, P = 0.39; Lax: 4.03 ± 0.40, P = 0.81; Clin: 4.20 ± 0.50, P = 0.61) relative to the baseline (4.03 ± 0.51). ICCs of the 3 readers for the comparison were 0.73 (95% CI [0.59, 0.83]) for banding artifacts, 0.80 (95% CI [0.69, 0.88]) for flow artifacts, and 0.62 (95% CI [0.42, 0.76]) for overall image quality. Figure 8 shows some demonstrative examples of the 5 groups when sequence parameters or the scanner was changed. Overall, the dual-stage network was able to achieve a similar performance in suppression of banding and flow artifacts compared with the baseline dataset. The VI sub-network also performed well in terms of artifact identification.

### Clinical evaluations

**Fig. 9:**
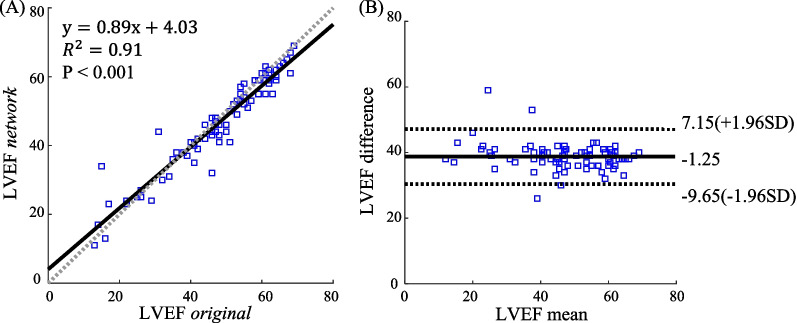
The correlation and difference between the LVEF evaluated using the original cine movies and that using the network-processed cine movies. The analysis was performed over 77 patients from the clinical dataset. There was a significant correlation between the two LVEFs (**A**). The difference between the two LVEF measurements was significant but very small (− 1.25%) and would usually not induce any difference for clinical diagnosis (**B**). LVEF, left ventricle ejection fraction

Over the 48 patients, the dual-stage network significantly improved banding artifact scores (4.01 ± 0.50 vs 2.77 ± 0.40, P < 0.001), flow artifact scores (4.22 ± 0.38 vs 2.97 ± 0.57, P < 0.001), and overall image quality (3.91 ± 0.45 vs 2.60 ± 0.43, P < 0.001) relative to the original cine (Additional file 3: Fig. S1). Furthermore, in 47 patients (97.92%), all 3 readers agreed that the VI map helps identification of the two types of artifacts. ICCs of 3 clinicians were 0.64 (95% CI [0.02, 0.85]) for banding artifacts, 0.82 (95% CI [0.29, 0.93]) for flow artifacts, and 0.71 (95% CI [0.06, 0.89]) for overall quality. These results suggest that the proposed network performed well in the clinical dataset, which was from a different scanner and a different cohort of patients. LVEF of the artifact-suppressed movies significantly correlated with that of the original movie (${R}^{2}$=0.91) in the 77 patients (Fig. 9). Bland–Altman analysis shows that the dual-stage network output led to a slightly reduced LVEF (mean bias = − 1.25%, P = 0.01).

### Processing time

The proposed technique takes less than a second on our server for processing a single cine movie loop.

## Discussion

In this work, we propose a partially interpretable dual-stage neural network for joint suppression of banding and flow artifacts in non-phase-cycled bSSFP cine. As a post-processing technique, the method reduces banding and flow artifacts relative to traditional cine without modifying the sequence. In addition, the proposed method does not provoke new flow artifacts due to the involvement of large frequency offsets, which is a problem for traditional full-range phase cycling. The VI stage of the network not only identifies where and which type of artifacts is present, but also explains why the network modifies the original image in the corresponding manner. In a busy clinical environment, clinicians may not have enough time to check for artifacts in every cine frame and slice. Owing to its partial interpretability and fast processing, the proposed network can be easily deployed in a clinical environment to both alert clinicians about the presence of artifacts and suppress them to improve the image quality.

Performance of the method in suppressing the two artifacts is largely driven by two factors in training of the network. Firstly, we developed a novel approach, which is the short-range phase cycling method, to obtain training labels. Prior to this work, how to jointly suppress banding and flow artifacts in bSSFP cine remains an open question. The challenge is that although phase cycling can well suppress banding artifacts, it also promotes flow artifacts in the image, which are difficult to completely suppress [14]. Several methods have been proposed, yet no one has been commonly used in practice [23]. Our data suggest that the proposed short-range phase cycling method can well address this issue. Another driver of the performance is the inclusion of 12 frequency offsets that densely cover the whole 2π range of the bSSFP spectral period [1]. Since each frequency is associated with different banding and flow artifact patterns, inclusion of them improves the diversity of the training data and generalizability of the method.

The artifact identification of the VI sub-network can be viewed as a “soft classification” task, where the label values are not binary but vary continuously between 0 and 1. Soft classification strategies have been also used in other computer vision tasks [33]. For our task, the adoption of $\mathrm{sigmoid}(\mathrm{SPC}\_\mathrm{label}/\mathrm{original}\_\mathrm{cine}-1)$ as a natural label for VI sub-network provides an objective and sensitive label for artifact identification. Consequently, the VI sub-network can even detect the zippers of the flow artifacts in the background, which is difficult to detect even by human observers. However, this label implicitly assumes that flow artifacts are always bright and banding artifacts are always dark. While this assumption is usually true, flow artifacts can also cause signal loss [16]. For those hypoenhanced flow artifacts, however, the network would consider them as banding artifacts, introducing a potential bias to the artifact identification.

An important observation from the results is that the network can only recover information based on the input image. If the artifacts occupy a large area in the image, the recovery of this area is an inference—much like what human observers would do in their mind—rather than a truthful reconstruction. Compared with phase cycling which obtains information from multiple frequencies, discrepancies may arise in those areas since phase cycling receives information that is invisible to the proposed method. Nevertheless, this problem may be resolved by combining e.g. two-fold phase cycling with a neural network. A standard linear combination from two-fold phase cycling may not result in satisfactory performance [5]. With the help of neural networks, this task can be more easily solved, so that both scan time can be reduced and reconstruction quality can be improved. In our results, we have also observed slight blurring, which is more evident in the abdomen and less in the heart. Potential causes of the blurring include the limitation of U-Net on preservation of fine-grained details [34], a lack of training data especially for the abdominal area, and the intrinsic blur in the SPC label due to a combination of multiple images acquired from different breath-holds. The use of more advanced architectures [34] or loss functions [35] and a collection of more training data may help to reduce the blurring. The slight blurring may explain the small LVEF discrepancy between the original cine and network-processed cine in the clinical dataset.

The unpaired, randomized evaluation of the network performance for different parameter variations confirmed that the method has a reasonable generalizability when sequence parameters or even the scanner is changed. While the performance was slightly reduced for certain parameter variations, such as the flip angle for band artifacts and slice thickness for flow artifacts, the performance reduction was within a reasonable range and did not significantly impair the image quality. The sensitivity of banding artifact suppression to a reduced flip angle may be explained by the poorer CNR and SNR. It is known that changes of image contrast can be a hurdle to generalization of deep learning models [36]. The reduction of slice thickness is known to increase flow artifacts [22]. An interesting finding is that the clinical dataset after processing by the proposed network had higher flow artifact scores than the baseline dataset. This may be due to a number of factors, such as the scanner difference, a better shimming, and subject characteristics. As many of the patients are elder people with cardiac dysfunction, their blood flow may be slower compared with young, healthy subjects, generating less flow artifacts.

## Limitations

Our study has limitations. Firstly, the qualitative clinical evaluation was performed in an unblinded fashion due to the apparent differences between regular cine and the network output, and the need to evaluate interpretability of the method. Although the readers were required to strictly comply with the criteria, potential bias may exist in the scoring. Secondly, the evaluation was based on data collected from a single vendor in two centers, which include a research institution and a clinical center. The sample size for clinical evaluation of the method was relatively small. Although the current sample size is sufficient to verify feasibility of the proposed method, generalizability of it to multi-vendor cine data collected from a larger cohort at multiple clinical centers awaits to be investigated.

## Conclusion

In conclusion, we propose a partially interpretable dual-stage neural network for joint suppression of banding and flow artifacts in bSSFP cine imaging. This post-processing method can reliably reduce banding and flow artifacts without a need for sequence modification, and provides artifact identity information to help interpretation of the results. The method can be potentially useful in a clinical setting to identify artifacts and improve image quality of bSSFP cine imaging.

### Supplementary Information

**Additional file 1: Document S1.** A supplementary description of the architecture of the dual-stage network, training details, and evaluation details.

**Additional file 2: Movie S1.** The original cine, VI sub-network output, and AS sub-network output for a cine movie acquired with a frequency offset of 82.5 Hz. Banding artifacts in the heart, abdomen, and subcutaneous fat regions, and flow artifacts in the heart and aorta regions were suppressed by the dual-stage network. VI maps generated by the VI sub-network explained each modification of the original image by the AS sub-network.

**Additional file 3: Figure S1.** Qualitative evaluation of the dual-stage network over 48 cine movies from the clinical dataset by 3 clinicians. The dual-stage network achieved improved banding artifact scores, flow artifact scores, and overall image quality relative to the original cine movie.

## Data Availability

The datasets used during the current study are available on reasonable request. The codes and trained models are openly available on GitHub (https://github.com/SJTU-CMRLab/Dual_stage_NN).

## Abbreviations

bSSFP: Balanced steady-state free precession
VI: Voxel identification
AS: Artifact suppression
FPC: Full-range phase cycling
SPC: Short-range phase cycling
BW: Reduced-bandwidth
FA: Reduced-flip-angle
ST: Reduced-slice-thickness
LAx: Long-axis
Clin: Clinical
LV: Left ventricle
LVEF: LV ejection fraction
CNR: Contrast-to-noise ratio
SNR: Signal-to-noise ratio
ROI: Region of interest
ICC: Intraclass correlation coefficient
