# Supplementary material for: A dual-stage partially interpretable neural network for joint suppression of bSSFP banding and flow artifacts in non-phase-cycled cine imaging
Source: J Cardiovasc Magn Reson. 2023 Nov 23;25:68. doi: 10.1186/s12968-023-00988-z (PMC10666342; doi:10.1186/s12968-023-00988-z)
Supplement: Supplementary file 1 — Additional file 1: Document S1. A supplementary description of the architecture of the dual-stage network, training details, and evaluation details. [file 12968_2023_988_MOESM1_ESM.docx]

**Additional File 1**

**Architecture of the dual-stage network**

***The VI sub-network***

The VI sub-network takes a single cine image as the input, and outputs a number at each voxel, which is valued near 1 if the voxel is in a dark band, near 0 if in a flow artifact, and near 0.5 if artifact-free. The VI sub-network uses U-Net as its backbone. Fig. 1A shows a schematic of the architecture. The U-Net architecture consists of four levels, each with two consecutive blocks in each limb and a skip connection between the two limbs. Each block includes a 3×3×3 three-dimensional convolution layer with a stride of 1 and a padding of 1, followed by a group normalization layer (the number of groups is 32) [1], and a ReLU activation function. Max-pooling is performed after each of the first 3 blocks in the encoding limb with a kernel size and stride of 1×2×2 (temporal×height×width). Max-unpooling is performed after each of the first 3 blocks in the decoding limb with the same kernel size and stride. The output layer consists of a 1×1×1 three-dimensional convolution layer followed by a sigmoid layer. The number of channels per layer is labeled in Fig. 1A.

***The AS sub-network***

The AS sub-network has two channels in the input layer. The first channel is fed with the original cine movie. The second channel is fed with the VI map generated by the VI sub-network. The AS sub-network simultaneously reduces banding artifacts and flow artifacts. The U-Net architecture of the AS sub-network is the same as the U-Net architecture of the VI sub-network, except for the output layer, which only includes a 1×1×1 three-dimensional convolution layer.

**Training details**

All cine images were interpolated, cropped, and normalized to enforce a uniform resolution of 1.5mm×1.5mm, image size of 192×192, and dynamic range of [0, 1]. Rigid image registration was performed between cine images of different center frequencies to correct potential motion between different breath-holds. Data augmentation was performed with two-dimensional image rotations, image translations, flipping, and elastic transforms [2]. The loss function for both VI and AS sub-networks was a linear combination of mean squared error and the perceptual loss [3,4]. The coefficients of this linear combination were manually optimized. Training was performed with ADAM with a learning rate of 0.0001. The batch size was 1. The network was implemented with PyTorch (version 1.11.0). Training was performed over 700 epochs for both sub-networks on a server equipped with a GPU (Tesla A100, NVIDIA, California, USA).

**Evaluation details**

For comparison of the proposed method with FPC, only the cine movies with a frequency offset of 82.5Hz were evaluated, as this frequency represents the usual level of frequency offsets encounterable during a practical scan. For evaluation of the partial interpretability, the analysis was performed in both end-diastolic and end-systolic phases for the frequency offset of 82.5Hz after removal of the background voxels. For evaluation of generalizability, 10 cine movies in each of the 6 groups were randomly chosen, generating a total dataset of 60 movies. The movies from healthy subjects had a frequency offset of 55 Hz, while those from patients had a 0Hz offset, since they were collected retrospectively. For the clinical evaluation, there were 48 patients whose cine movies received qualitative evaluation. Among the 48 patients, 16 were evaluated based on their short-axis movies, 16 based on their two-chamber movies, and 16 based on their four-chamber movies after randomization. For evaluation of banding artifact suppression, flow artifact suppression, and overall image quality, the scores from the 3 clinicians were averaged before the final assessment. For evaluation of the partial interpretability, the minimal score of the 3 clinicians was used for the final assessment; that is, the final score was 2 only when all 3 readers scored 2. For quantification of the LVEF, the software SEGMENT [5] was used without any manual intervention.

**References**

1. Wu Y, He K. Group Normalization. 2018. p. 3–19. Available from: https://openaccess.thecvf.com/content_ECCV_2018/html/Yuxin_Wu_Group_Normalization_ECCV_2018_paper.html

2. Simard P, Steinkraus D, Platt J. Best practices for convolutional neural networks applied to visual document analysis. Icdar. 2003;

3. Simonyan K, Zisserman A. Very Deep Convolutional Networks for Large-Scale Image Recognition [Internet]. arXiv; 2015. Available from: http://arxiv.org/abs/1409.1556

4. Dosovitskiy A, Brox T. Generating Images with Perceptual Similarity Metrics based on Deep Networks. Adv Neural Inf Process Syst [Internet]. Curran Associates, Inc.; 2016. Available from: https://proceedings.neurips.cc/paper/2016/hash/371bce7dc83817b7893bcdeed13799b5-Abstract.html

5. Berggren K, Hedstrom E, Ehrenborg KS, Carlsson M, Engblom H, Ostenfeld E, et al. Multiple Convolutional Neural Networks for Robust Myocardial Segmentation.
