# Supplementary figures and images for: A dual-stage partially interpretable neural network for joint suppression of bSSFP banding and flow artifacts in non-phase-cycled cine imaging
Source: J Cardiovasc Magn Reson. 2023 Nov 23;25:68. doi: 10.1186/s12968-023-00988-z (PMC10666342; doi:10.1186/s12968-023-00988-z)

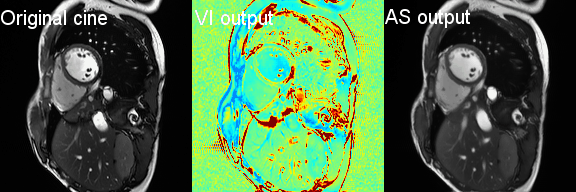

Supplement: Supplementary file 2 — Additional file 2: Movie S1. The original cine, VI sub-network output, and AS sub-network output for a cine movie acquired with a frequency offset of 82.5 Hz. Banding artifacts in the heart, abdomen, and subcutaneous fat regions, and flow artifacts in the heart and aorta regions were suppressed by the dual-stage network. VI maps generated by the VI sub-network explained each modification of the original image by the AS sub-network. [file 12968_2023_988_MOESM2_ESM.gif]
