# Supplementary material for: A dual-stage partially interpretable neural network for joint suppression of bSSFP banding and flow artifacts in non-phase-cycled cine imaging
Source: J Cardiovasc Magn Reson. 2023 Nov 23;25:68. doi: 10.1186/s12968-023-00988-z (PMC10666342; doi:10.1186/s12968-023-00988-z)
Supplement: Supplementary file 3 — Additional file 3: Figure S1. Qualitative evaluation of the dual-stage network over 48 cine movies from the clinical dataset by 3 clinicians. The dual-stage network achieved improved banding artifact scores, flow artifact scores, and overall image quality relative to the original cine movie. [file 12968_2023_988_MOESM3_ESM.docx]

**Additional File 3**


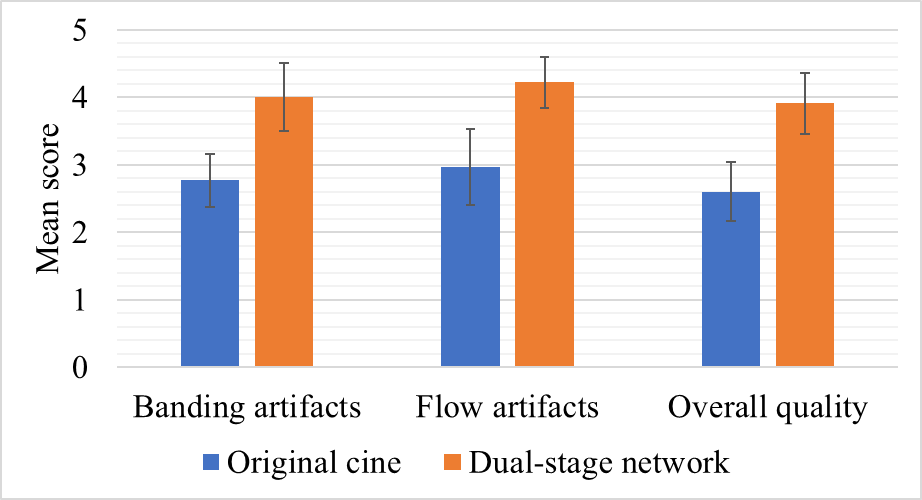


***

***

***

**Fig. S1** Qualitative evaluation of the dual-stage network over 48 cine movies from the clinical dataset by 3 clinicians. The dual-stage network achieved improved banding artifact scores, flow artifact scores, and overall image quality relative to the original cine movie.
